# Understanding health advocacy in family medicine and psychiatry curricula and practice: A qualitative study

**DOI:** 10.1371/journal.pone.0197590

**Published:** 2018-05-23

**Authors:** Sophie Soklaridis, Carrie Bernard, Genevieve Ferguson, Lisa Andermann, Mark Fefergrad, Kenneth Fung, Karl Iglar, Andrew Johnson, Morag Paton, Cynthia Whitehead

**Affiliations:** 1 Department of Education, Centre for Addiction and Mental Health, Toronto, Ontario, Canada; 2 Department of Psychiatry, Faculty of Medicine, University of Toronto, Toronto, Ontario, Canada; 3 Department of Family and Community Medicine, Faculty of Medicine, University of Toronto, Toronto, Ontario, Canada; 4 Ethnocultural Assertive Community Treatment Team, Mount Sinai Hospital, Toronto, Ontario, Canada; 5 Department of Psychiatry, Sunnybrook Health Sciences Centre, Toronto, Ontario, Canada; 6 Asian Initiative in Mental Health Clinic, University Health Network, Toronto, Ontario, Canada; 7 Continuing Professional Development Office, Faculty of Medicine, University of Toronto, Toronto, Ontario, Canada; 8 The Wilson Centre, Toronto, Ontario, Canada; 9 Health Professions Research, University Health Network, Toronto, Ontario, Canada; 10 Department of Education, Women’s College Hospital, Toronto, Ontario, Canada; Brown University, UNITED STATES

## Abstract

**Background:**

We explored understanding and experiences of health advocacy among psychiatry and family medicine residents and faculty and the implications for clinical care and teaching through the lens of relationship-centred care.

**Methods:**

This qualitative study was conducted in the psychiatry and family medicine departments at a large urban university. We interviewed 19 faculty members and conducted two focus groups with 18 residents. Semi-structured questions explored the relational meaning of health advocacy, how residents and faculty learned about the role and ethical considerations involved in incorporating advocacy work into clinical practice within a relationship-centred care framework.

**Results:**

Four themes emerged from the interviews and focus groups: 1) health advocacy as an extension of the relationship to self; 2) health advocacy and professional boundaries in the physician–patient relationship; 3) health advocacy within a team-based approach; and 4) health advocacy and the physician–community/organization relationship. Participants described implications for practice of the challenges of health advocacy, including perceived institutional risks, professional boundaries and the appropriation of patient voice.

**Conclusions:**

Our study provides insights into the relational complexities of the health advocate role in residency curriculum and clinical practice. All participants described health advocacy as a broad spectrum of actions that are guided by relationships among patients, health care professionals and communities. Our analysis revealed that some challenges that participants identified with a health advocacy role could be addressed by anchoring the role within a specific theoretical framework. This would better enable us to create a culture of advocacy in the training and development of physicians.

## Introduction

In Canada, health advocacy is a core competency that is recognized by medical education and regulatory bodies [1,2]. The Canadian Medical Education Directions for Specialists describe the health advocacy role: “physicians responsibly contribute their expertise and influence to improve health by working with the patients, communities, or populations they serve to determine and understand needs, develop partnerships, speak on behalf of others when needed, and support the mobilization of resources to effect change” [3].

It is widely agreed that the definition of health advocacy and the expectations at each level of training need more clarity and direction [4]. Oandasan [5] calls for an “operational definition” that would enhance health advocacy teaching methods; others emphasize the difficulty in teaching this role, in particular, and the resulting need for curriculum and faculty development [6–8]. There is also ongoing debate about whether physicians have a moral, social and professional responsibility to address health care issues beyond the direct care of their patients [9, 10].

Family medicine and psychiatry are the medical disciplines that disproportionately work with marginalized and underprivileged segments of the Canadian population (or at least whose work must recognize the effects of marginalization to provide effective care). Developing health advocacy skills, therefore, is an important focus for residents and physicians in both disciplines. the current study provides an interesting opportunity to examine similarities and differences between psychiatry and family medicine approaches.

There is a clear need to explore the range and scope of health advocacy in clinical education and practice [11]. At the time of this study, neither of the two residency programs offered a formal curriculum focused on health advocacy, and anecdotal evidence suggested that the concept of health advocacy did not easily fit within traditional models of medical education. The aim of this qualitative, exploratory study was to examine understanding and experiences of health advocacy among family medicine and psychiatry residents and faculty within the context of curricula and practice.

## Methods

### Setting

This study was conducted between October 2014 and October 2015 with the Department of Psychiatry and the Department of Family and Community Medicine at a large urban Canadian university. These departments train more than 644 residents and have over 1,500 faculty members across 22 training sites [12].

This study was approved by the Centre for Addiction and Mental Health Review Ethics Board and the University of Toronto Review Ethics Board.

### Study design

This exploratory qualitative research study used a thematic analysis approach [13]. The approach involves an interactive process in which a shared understanding of the phenomenon is explored between the researcher and the study participant [14]. The researcher asks participants questions and may pursue particular areas that emerge, and participants can ask questions or respond to questions in a creative and flexible manner to ensure that their perspective is fully described or explained. Our research question was: How do residents and faculty in family medicine and psychiatry experience and conceptualize health advocacy in both curricula training and clinical practice?

We were particularly interested in how relational dimensions (HCP/resident relationship to self, patient, one another and their communities, both public and professional) influence how faculty members and residents conceptualize health advocacy.

### Recruitment, sampling and participants

Purposeful, convenience and snowball sampling techniques were used to identify potential participants [15]. We chose to recruit participants from the Department of Psychiatry and the Department of Family and Community Medicine because we had established partnerships early on with research team members from both departments. The psychiatrists and family medicine physicians on our team had different ideas of how advocacy was conceptualized in their clinical practice, and we were interested to see whether the differences would emerge during data collection. However, because the focus of the study was not on examining differences in how the disciplines of psychiatry and family medicine conceptualize advocacy work, any differences we note are simply for the purpose of identifying future research directions. Using purposeful sampling, we began by inviting faculty members directly involved in planning and implementing the curriculum, as well as residents with experience in the curriculum who would have a perspective on the research question. We asked administrators to identify residency directors or coordinators who might be interested in participating in the study. We also asked resident leaders (i.e., chief residents) for the contact information of residents who might be interested in participating in the focus groups. Convenience sampling involved an e-mail to the Office of Education Scholarship in the Department of Family and Community Medicine; an e-mail to the Psychotherapies, Humanities and Education Scholarship through the Department of Psychiatry listserv; and e-mails through the departmental postgraduate medical education offices. Snowball sampling involved asking participants after each interview or focus group to identify other potential participants.

Recruitment was conducted mainly through e-mail and in-person communication. One of the co-investigators sent an e-mail to site directors and other faculty that provided a brief overview of the study (title, research team, summary). Those who were interested replied to the research analyst, with whom they determined a convenient time for the interview. Participants were not offered incentives for participation. We conducted 19 one-on-one semi-structured interviews with faculty, and 18 residents participated in the focus groups.

### Data collection

Qualitative data were collected through the faculty interviews and resident focus groups. In the interviews, faculty members were invited to share their personal stories, including difficulties with teaching and implementing health advocacy into their clinical practice, and their interpretation of these challenges. All interviews remained confidential. The focus groups examined how residents formed their thoughts and opinions as a group. The social interactions generated by this type of group yield rich data [16]. The current study used focus groups to explore the overall culture, group experiences and attitudes toward health advocacy among residents [17].

The research analyst and third author (GF) conducted nine interviews with psychiatry faculty and 10 interviews with family medicine faculty. There were nine female and 10 male faculty participants. The research analyst held one focus group with psychiatry residents (n = 12) and another with family medicine residents (n = 6). There were 13 female and five male resident participants.

The development of the initial interview questions and focus group guides reflected both the authors’ experiences and themes identified in the literature on the role of health advocates in medical education. The questions were modified as patterns emerged throughout the data collection process [18]. The following questions were asked in both interviews and focus groups:

- What does being a health advocate mean to you?
- How do we learn to be health advocates?
- What are the ethical considerations/challenges of health advocacy?

Most interviews lasted 30 to 60 minutes, and focus groups lasted between one and one and a half hours [18].

### Data analysis

We used an iterative and constant comparative method that allowed the researchers to reduce the data through constant recoding [19]. The researchers thematically analyzed all of the content from the interviews and focus groups together to explore how health advocacy is experienced and conceptualized by residents and faculty in family medicine and psychiatry in both curricula and clinical practice [20, 21]. Although the content was treated as one dataset, we identified the discipline and whether the comments were made by residents or faculty members, which allowed us to note differences between disciplines. The audio-recordings were transcribed verbatim by a professional transcriptionist. We held three coding and analysis meetings. During the first meeting, four authors (SS, CB, MP, GF) developed a coding dictionary based on three transcripts. In the second meeting, thematic categories were developed from the codes that emerged from the data and NVivo software was used to organize the data. In the third meeting, we categorized the data into themes and, through consensus, refined the analysis of the findings. This transparent and iterative process ensured that the rigour and trustworthiness of the data were maintained. The codes, themes and subthemes were formulated and modified throughout the entire research process with the full participation of all researchers. Our diverse backgrounds informed the analysis and ensured that no single perspective prevailed [22].

Since we were interested in the relational aspects of health advocacy, we applied the lens of relationship-centred care. Relationship centred care is a framework based on the following principles: personhood matters; affect and emotions are important; relationships do not occur in isolation; and maintaining genuine relationships is necessary for health and recovery [23, 24]. The relationship-centred care framework is useful because many types of relationships exist in health care. These include the relationships that physicians have with their patients, their colleagues, their community or organization and with themselves. In an educational environment, there is also the relationship that physicians have with their students or residents. We explored how health advocacy was shaped by self-awareness and knowledge and maintained through physicians’ connection to patients, other colleagues and the larger community or organization. Through this lens, we wanted participants to also reflect on the relational barriers and challenges they faced when engaging in advocacy work. To our knowledge, this is the first study that captures the in-depth experiences and perceptions of both residents and faculty in psychiatry and family medicine regarding the relational dimensions of health advocacy.

## Results

All participants experienced and conceptualized health advocacy as an interpersonal process that sees relationship-building as central to improving patients’ health. They also described the challenges of health advocacy work, which include perceived institutional risks (being disciplined by supervisor; termination), issues related to professional boundaries and the appropriation of patient voice.

Participants’ responses were categorized into four relational themes:

1. health advocacy as an extension of the relationship to self
2. health advocacy and professional boundaries in the physician–patient relationship
3. health advocacy and the team-based approach
4. health advocacy and the physician–community/organization relationship.

Although a comparative analysis of responses by discipline/department was not planned for this study, we did identify elements that seemed to be unique to each discipline/department with regards to health advocacy. These differences may be a worthwhile focus for future research.

### Health advocacy as an extension of the relationship to self

Several participants linked health advocacy to one’s self-identity and values. Most participants believed that students come to medical school as advocates, often with extensive volunteer experience and a deep understanding of the social determinants of health. As one psychiatry resident described:

I saw myself as an activist before I entered medical school… Part of the reason I went into medicine was I could see the possibilities for connecting those dots. For me… the lens you look through and the perspectives that you take is always influenced by these fundamental values.

Several participants discussed how personally rewarding advocating for patients can be. One family medicine faculty member explained:

When you do something that feels a little bit beyond the call of duty… and it actually makes a difference. [For example, when] the family comes back later and says, “That letter that you wrote to the school really helped.”

These comments reflect an overall sense that advocacy emanates from an authentic part of one’s personhood. It is intricately tied to one’s experiences and identity as a physician, and it is rewarding.

### Health advocacy and professional boundaries in the physician–patient relationship

The focus of discussions about professional boundaries in advocacy work within the physician–patient relationship differed between psychiatry and family medicine participants. Most psychiatrists described the need to be mindful of boundaries from an empowerment perspective. They were concerned about disempowering patients by speaking *for* not *with* patients. In the words of one psychiatrist faculty member:

I don’t like this idea of physicians speaking for other people. I think that’s often the role we’re put in, it’s what we’re asked to do. You have this power and expertise and influence; now use it on behalf of somebody.

Psychiatry participants were also concerned about perpetuating power imbalances and hierarchies when speaking from a position of privilege, as one psychiatry resident explained:

As a physician, you shouldn’t be advocating for people in a way that just continues to let you, as someone already privileged, have a voice and just always speak for someone on their behalf.

Family medicine participants, on the other hand, did not focus on power and privilege when they talked about professional boundaries. Instead, they emphasized the importance of maintaining professional boundaries in advocacy work to prevent burnout. One family medicine resident described this challenge:

I’ve had people say things like “You're getting too invested, you need to let go.” Which I think is people’s attempt at making [residents] have realistic expectations. They don’t want me to go home and cry at night because of a patient.

Both family medicine faculty and resident participants identified burnout as a critical issue for the profession, in general, and for health advocates, in particular. However, the issue was not widely discussed by psychiatry resident and faculty participants.

### Health advocacy and the team-based approach

Participants described the importance of a team-based approach to health advocacy. They attributed their success in advocacy work to their relationships with colleagues and to partnerships in the community, As one psychiatry resident described:

Part of involving yourself in advocacy is… attaching yourself to a team…, seeking out mentors, speaking to social workers and not isolating yourself… at least being aware of what the system [has to offer]… Because that’s your gateway to advocacy. You can’t do this on your own.

In terms of formal education, most participants agreed that there is a need to better integrate health advocacy into the existing curricula, especially as a way to promote interprofessional collaboration. Since health advocacy is rarely included in the formal didactic curriculum, many participants emphasized the importance of role modelling in helping residents to develop this competency for medical practice. One psychiatry faculty member highlighted this need:

If you don’t practise something as a resident and you don’t see it validated, if the hidden curriculum is “a doctor is not really an advocate, leave that for the social worker,” then it doesn't matter what we say. [Residents] have to see faculty that they respect doing it [advocacy].

Many faculty participants thought that role modelling and implicit learning through observation may not be enough, and that acts of health advocacy need to be taught explicitly. Ordinary acts of advocacy are often overlooked; these are actions that go beyond identifying and treating illness to contribute to the broader well-being of patients. One family medicine faculty participant suggested that educators tell their residents, “I’m advocating for this person in this way, and I tell them about these social services in this way,” which leads to discussion and deliberate reflection of who else on a health care team can be involved in the patient’s care.

### Health advocacy and the physician–community/organization relationship

Participants identified community outreach and raising awareness about systemic issues, such as refugee health and poverty, through activism as components of health advocacy work. As one psychiatry faculty member pointed out:

Psychiatry can be very individualistic and based on identifying and treating and problematizing aspects of the individual. But in an outreach context, you quickly realize that you can’t do much just focusing on that. You have to understand the larger community and social context.

For several participants, their understanding of the importance of advocacy grew through personal connections to the community. As one psychiatry faculty member explained:

I have a very vivid memory at the end of the [school] term, they had some school fair and I remember they had this pie-eating contest. I had my face with cream all over it. The kids loved it to see a doctor looking like a fool. And I think that sort of reflected that I was part of that community and I think it did help me advocate for those kids.

However, there was no consensus among all participants about how best to advocate at the system level when health system issues become politicized. One family medicine faculty member, for whom advocacy includes political activism, described an experience with a small group of like-minded physicians:

Some fellow doctors… are very upset with how we [activists] behave, disrupting cabinet announcements, occupying a [politician]’s office…. People think it’s impolite and improper.

Some psychiatry faculty participants expressed fear of reprimands by their institution for taking part in public activism as health advocacy work. One psychiatry faculty member expressed this concern:

I’ve been in situations where I've thought, “Am I going to get in trouble professionally if it comes out that I am doing this [attending protests and rallies]?”And I think… in some sense you’re more vulnerable because you’re affiliated with this institution.

Unlike psychiatry participants, family medicine residents and faculty did not share this fear of institutional reprimands for participating in public activism.

At the curricular level, health advocacy was a concept that all participants identified as being least spoken about in residency training. There was concern that formalizing health advocacy in the didactic curriculum would mean compromising residents’ ability to learn other competing competencies in the time-constricted curriculum. A few participants also raised philosophical concerns. As one psychiatry resident explained, formalizing the advocacy role in the curriculum risks losing what lies at the heart of advocacy:

[A]dvocacy is founded on something that you are fundamentally interested in or interested in changing. I think if you take that element out and make it formalized …then that takes [out] the personal attachment to whatever it is that you’re advocating for and therefore you're not going to advocate for it as strongly as you would if it was more of a natural process.

One strategy for balancing health advocacy within an already challenging curriculum could be to create opportunities for residents to conduct academic projects based on advocacy, as one family medicine faculty member suggested:

We have an academic project going on right now with the residents around poverty and resources for poverty and assessing for poverty. So it triggered me that we could encourage our residents to do more formal projects around advocacy specifically. They already do a quality project, they do an academic project, but not all of them see advocacy as a type of project they could be doing as an academic project.

Participants agreed that there is a need to assess how health advocacy is taught in the family medicine and psychiatry residency curricula. However, participants did not offer concrete suggestions for how to implement assessments.

## Discussion

Health advocacy has been described as a “time-honoured role for physicians around the globe,” enacting a part of physicians’ social responsibility to promote the health of individuals, communities and populations [25]. Although participants deemed advocacy to be essential to their clinical practice, the parameters of “approved” or “authorized” forms of advocacy remain unclear. Our study provides insights into the relational complexities of the health advocate role in residency curriculum and clinical practice.

Participants described health advocacy in terms of relationships. A relationship-centred care framework begins with recognizing that each clinical encounter is a convergence of multiple, distinct perspectives: that of the patient, the physician, the patient’s family and the community. Reflecting on these diverse perspectives and the values they embody is critical to advocacy work. How health advocacy is delivered and received depends on how we define ourselves and others within multiple relationships, acknowledging power differentials and sociocultural contexts.

Although most participants in our study identified as health advocates, they raised issues that affect their ability to engage in advocacy work while staying true to their values and beliefs. Participants shared experiences of personal and professional discomfort with what it means to be a health advocate in residency and clinical practice.

It is well understood in the medical education literature that values underpin all clinical decision making [26]. The absence of an ethics-based framework [27] can create a sense of moral burden and distress among physicians who are trying to balance these multiple roles and accountabilities. The process by which health system leaders set priorities and make decisions about resource allocations has also been examined [28]. Health care leaders have been described as “jugglers” and “tightrope walkers” who are accountable to a variety of “ringmasters” [28]. This framework provides steps and “value filters” that can support physicians at the individual and organizational level around reflecting on ethical considerations in promoting fairness while reducing subjective decision making and the moral burden associated with advocacy work. Infusing an ethics and values-based approach in health advocacy could lessen the burden and ambiguity of this role. The literature also describes humanistic care as a mechanism for increasing collective consciousness in the culture of medicine [29]. Identifying the culture and core shared values within medicine can begin to inform and clarify health advocacy in residency training and clinical practice.

Interesting differences between the disciplines of psychiatry and medicine emerged in this study that could affect how willing physicians are to engage in advocacy work. Psychiatry faculty members feared reprimands from their institution for taking part in public activism, but family medicine participants did not share this concern. The difference might be due partly to the increased sensitivity of psychiatrists to stigma and to controversy about the specialty. Many family medicine faculty in our study practised in community hospital settings, whereas most psychiatry faculty practised in academic health science centres, which might have policies that govern how physicians advocate publicly on behalf of patients or the health care system. As one family physician put plainly, “sometimes speaking out has a price” [25]. Physicians have been blacklisted or lost their jobs as a result of their health advocacy work [25]. The Canadian Medical Protective Association suggests that physicians working in hospitals notify hospital administration before engaging in advocacy activities because their activities could be misconstrued as representing the hospital [30]. At the same time, the Canadian Medical Association states that physicians “must be able to freely advocate when necessary on behalf of their patients and should do so in a way that respects the views of others and is likely to bring about meaningful change that will benefit their patients and the healthcare system” [31]. While these views support physicians in their role as health advocates, they also highlight the nuances and challenges of practising and teaching this role. How these challenges play out in clinical practice could be useful information to both curriculum and faculty developers as they continue to support and promote the integration of health advocacy into medical education.

Our findings are preliminary and are most useful as a set of hypotheses to be tested more rigorously in future studies. The findings should be interpreted in the context of several limitations. First, because only residents and physician faculty were interviewed, we may not have identified themes that reflect the perspectives of other health care disciplines, such as social work and nursing, as well as patients and families. Second, while some differences between psychiatry and family medicine participants were noted, other factors, such as the context in which physicians practice, might account for these differences. Future research that explores the historical and social contexts of the two disciplines could help us to understand why the two disciplines differ in their approach to and perspective on advocacy. Finally, research about how to assess health advocacy in the residency curriculum and clinical practice is also needed.

## Conclusions

Residents and faculty in family medicine and psychiatry experience and conceptualize health advocacy in terms of its relational complexities in both curricula training and clinical practice. They believe that health advocacy is essential and rewarding, but are also concerned about challenges associated with advocacy work, including perceived institutional risks, professional boundary issues and appropriation of patient voice. Making ethical decisions and judgments are a fundamental part of the daily work of physicians, so there needs to be a clear ethics and value-based framework in place that accounts for the diverse perspectives of all stakeholders. This proposed framework would outline difficult ethical situations and potential solutions. We encourage future research to build on the results of this study. The growing body of evidence will help medical educators and planners to develop training that supports residents and new health care practitioners in doing health advocacy work.
